# Consumer and Stakeholder Perspectives About the Lived Experience Advisor Programme: A Qualitative Descriptive Study

**DOI:** 10.1111/hex.70831

**Published:** 2026-08-21

**Authors:** Rebecca Barbara, Rohan Vaughan, Joanna Petrunic, Debra Kerr

**Affiliations:** ^1^ Western Health St Albans Victoria Australia; ^2^ School of Nursing & Midwifery, Centre for Quality & Patient Safety Research in the Institute for Health Transformation Deakin University, Geelong Victoria Australia

**Keywords:** advisor, consumer, evaluation, lived experience, perspectives, qualitative descriptive study

## Abstract

**Background:**

The Lived Experience Advisor Program (LEAP) was introduced as a new approach to consumer engagement within a large healthcare organisation. The programme was designed to professionalise and embed lived experience as a recognised form of expertise within organisational decision‐making, research, and quality improvement through formal paid employment.

**Objectives:**

The aim of this study was to explore Lived Experience Advisor and stakeholder perspectives about the LEAP.

**Methods:**

A qualitative descriptive study was conducted within the metropolitan health service in which the programme was initiated. Data was collected through audio‐recorded, individual, semi‐structured interviews from May to June 2025. Data analysis was undertaken using a thematic content analysis approach.

**Study Participants:**

Consumers employed as Lived Experience Advisors (*n* = 7) within the study organisation, and stakeholders who utilised the LEAP (*n* = 7), participated in this study.

**Results:**

Fourteen individuals participated. Since inception, Lived Experience Advisors have contributed to projects in youth health services, carer support, emergency care, and development of consumer engagement strategies. Three major themes emerged: (1) preparation for the lived experience advisor role, (2) valued contributions to organisational projects, and (3) restrictions on role influence. Comprehensive orientation and training are integral to the successful integration of Lived Experience Advisors as health service employees. Role clarity could be strengthened across the organisation. Participants described the value of organisational commitment to build consumership, with tangible benefits for various projects and individual growth. Components of the LEAP could be modified to maximise influential opportunities for Lived Experience Advisors.

**Discussion:**

Employment of Lived Experience Advisors within the healthcare organisation across a range of clinical and operational projects has enhanced service planning and health literacy for diverse communities. Building a community of practice is needed to strengthen mentorship and professional development needs for Lived Experience Advisors.

**Conclusions:**

The contribution of consumership to healthcare has been realised through implementation of the LEAP. Consideration is needed regarding working conditions to sustain retention and promote career progression.

**Patient or Public Contribution:**

Consumers who were employed as Lived Experience Advisors within the study organisation provided valuable feedback regarding the study design. They were also invited to participate in the study.

## 1 Introduction

In contemporary healthcare reform, there is increasing recognition that people with lived experience of health conditions requiring frequent hospitalisation should be actively involved in the design of services and interventions tailored specifically for them [1]. This has led to a shift from traditional, clinician‐led models toward ‘co‐design’ in which health service providers integrate experiential knowledge with clinical expertise to create solutions that are practical and effective [2]. Growing evidence was identified in a systematic review of 23 randomised controlled trials by Wiles et al. [3] for the positive effects of consumer engagement on the relevance and outcomes for health policy, research and services. Most effective engagement strategies incorporated consumer empowerment processes in the development and implementation phases of the improvement initiative. A recent Cochrane review was undertaken to assess the effects of consumers and health providers working in partnership to promote person‐centred health services. Only five studies were identified, indicating a lack of robust evidence. They proposed that qualitative research is needed to build the evidence base to determine the effects of consumers and providers working in partnership to plan, deliver or evaluate health services.

There is widespread acknowledgement that incorporating consumers' opinions in the development and improvement of healthcare is important. Traditionally, consumer involvement has centred on two models: 1) representation on advisory councils, boards and committees; and 2) patient satisfaction or experience surveys [4]. The Lived Experience Advisor Program (LEAP) was introduced into the study organisation in 2023 as a novel approach to enhance consumer leadership throughout the organisation [5]. People with lived experience as a consumer or carer are formally employed as Lived Experience Advisors (LEA) on a part‐time basis, averaging 1 day per week. The primary focus of their role is to ensure consumer insight is applied in project/research work, through improved health literacy and service navigation, and ensuring outcomes are accessible, culturally appropriate and safe.

LEAs are recruited across a wide range of projects, drawing on their lived experience as consumers or carers of people with health conditions, such as dementia, substance addiction, and cancer. They may also be engaged as members of diverse communities (e.g., cultural, gender and neurological diversity) and Aboriginal and Torres Strait Islander peoples. The operations manager who holds the portfolio of ‘Best Experience’ within the study organisation formally advertises vacant positions through standard employment processes. Individuals are interviewed and selected based on selection criteria and project demand. Following acceptance of the appointment, including a formal signed contract, LEAs are expected to complete formal induction, such as workplace health and safety, cultural awareness, and other orientation training. As identified, additional self‐directed informal learning is provided to improve individuals' core capabilities such as project management, change management, and stakeholder engagement. They also have access to teaching and learning programmes available to all new employees.

LEAs are supervised by the operations manager, including informal regular meetings and performance appraisal. There is an expectation that LEAs manage their workload to meet project demands, in consultation with the operations manager. Flexible work arrangements are encouraged to enable LEAs to have control over their work commitments (e.g., remote work, flexi‐hours) and personal responsibilities. They are provided a desktop computer, office space, and dedicated email account within the health organisation. Accessibility needs are addressed on an individual basis. For example, a Disability Liaison Officer is employed to work alongside an LEA with a cognitive impairment in all project meetings.

Whilst the organisation has an extensive voluntary network which exceeds 180 people, the LEAP complements and enhances existing consumer engagement structures by elevating consumer leadership and building co‐design practice capability. The driving purpose of the LEAP was to integrate LEAs into key decision‐making processes and improvement initiatives, ensuring that consumer voices are not just heard but actively shape the design and delivery of services within the organisation Hall et al. [4]. propose that there is a need for an active and comprehensive model of consumer involvement to enhance patient‐centred care and influence health system improvement. Several components of the LEAP reflect the model of consumer involvement proposed by Hall et al. [4] including a selection process, focused training, regular meeting attendance, and support through a peer‐based, online, Community of Practice (CoP). At regular monthly intervals, LEAs are invited to attend an online meeting which facilitates discussion and shared learning in a safe environment.

The role of consumers has evolved significantly in the past 20 years in health care. Health services are expected to actively integrate lived experience into governance, research, and improvement projects to promote safe, high‐quality health care [6]. Consumer perspectives should be incorporated at the direct care, service and systems levels. However, consumer involvement remains predominantly voluntary, with inconsistent levels of engagement.

There is limited evidence regarding programmatic approaches to strengthen consumer involvement in healthcare quality improvement initiatives in hospital settings. Most research describes consumer involvement in outpatient or community settings [3]. Due to the broad range of projects and roles, it is not feasible to evaluate the impact of the LEAP using formal outcome measures. Hence, this study was designed to explore LEAs' and stakeholders' opinions about the LEAP.

## 2 Methods

### 2.1 Aim and Study Design

The aim of this study was to explore Lived Experience Advisor and stakeholder perspectives about the LEAP. This was a qualitative descriptive study undertaken through individual audio‐recorded interviews. This methodology was selected because it facilitates a descriptive, non‐interpretive exploration of participants' perspectives [7]. Qualitative description is a methodology that can be used to describe data in the participants' own words, providing insight into events experienced by the participants. The Consolidated Criteria for Reporting Qualitative Research (COREQ) checklist [8] was followed in preparation of this manuscript (refer to Supplement 1).

### 2.2 Study Participants

Participants included LEAs and stakeholders who had previously utilised the LEAP in a project, research and/or evaluation activity. At the time of the study there were seven LEAs formally employed within the study organisation. Participants received an incentive upon completion of the interview: LEAs were offered a $A50 online voucher, while stakeholders received a $A10 voucher for a local café. Increased incentives were provided to LEAs because interviews were conducted outside their employment hours. In contrast, stakeholder interviews were performed within their employment hours.

### 2.3 Sampling Strategies

A convenience sampling approach was used. LEAs and stakeholders were invited to participate via email, which included an attachment with written information regarding the study's purpose, procedures, and voluntary participation. Interested individuals contacted the Principal Investigator directly to organise an interview.

Sample size was determined by data saturation, defined as the point at which no new information emerges during analysis and further data collection is unlikely to add value [9]. A minimum target of five LEAs and five stakeholders was set. Data saturation was considered achieved after ten interviews.

### 2.4 Data Collection

Data were collected through individual, semi‐structured interviews. An interview guide comprising open‐ended questions was developed in consultation with one consumer, employed as an LEA (Table 1).

**Table 1:** Interview guide.

**Lived experience advisors**	
1	How did you hear about the programme?
2	Can you tell me about the orientation you received for the role? In your view, was it comprehensive enough?
3	In your role as an LEA^1^, what activities have you enjoyed the most/least?
4	Have you experienced any barriers when employed as an LEA in a project?
5	What could be improved in the LEA programme?
6	Are there other areas you think you could be involved in as an LEA?
7	Do you feel supported in the LEA role?
8	What would encourage you to continue in the LEA role?
9	How does the LEA role differ from other consumer engagement roles you might have held before or consumer activities you have done before?
**Stakeholders**	
1	How did you hear about the programme?
2	Were you provided an explanation of the LEAP2?
3	In what ways have you utilised the LEAP?
4	What aspects of the LEAP have you benefited the most?
5	What aspects of the LEAP have you enjoyed the least?
6	What was the most successful project that included involvement of an LEA?
7	What was the least successful project that included involvement of an LEA?
8	How well did LEAs integrate into the team?
9	What could be improved to the LEAP?
10	Are there other projects in your service area that LEAs could be involved in?
11	Have you felt supported when utilising LEAs?
12	What would encourage you to continue employing LEAs in projects in your area?
13	Have you experienced any barriers to LEAs being actively involved in your projects?
14	In what way could this role be expanded?

Abbreviations: LEA, lived experience advisor; LEAP, lived experience advisor programme.

All interviews were conducted by one researcher (JP), a female registered nurse with prior interviewing experience. No prior relationship was established prior to interviews. In‐person and telephone interviews were audio‐recorded using a digital recorder, and online interviews were recorded using Zoom. For in‐person data collection, interviews were conducted within the study organisation. A Disability Liaison Officer was present during the consent and interview processes for one participant with cognitive impairment.

Audio recordings were transcribed verbatim by a professional transcription service (Pacific Transcriptions). Transcripts were not provided to participants for validation; two researchers confirmed the accuracy of the transcriptions. Transcripts were uploaded to NVivo for analysis and assigned a unique identifier (e.g., ‘LEA1’, ‘SH1’) in the order interviews were conducted.

### 2.5 Data Analysis

Data analysis was conducted using Braun and Clarke's [10] six‐phase thematic analysis approach. Preliminary data analysis was undertaken following interviews of five stakeholders and five LEAs using an iterative, data‐driven approach. Similar themes were identified across, and within, the datasets. Hence, a decision was made to analyse the data collectively. To confirm these findings, a further two individuals from each participant group were interviewed.

To reduce potential biased interpretation, the two researchers (JP, DK) who held no responsibility for the LEAP independently read all transcripts to familiarise themselves with the data and documented initial ideas. Each researcher then reviewed transcripts line‐by‐line, assigning preliminary codes and allocating illustrative quotes to potential themes and sub‐themes. Continuous refinement of the codes ensured that coding was responsive to the data. Following independent coding, emergent themes and sub‐themes were established in two separate consensus meetings. A final thematic framework was refined in a final study team meeting once consensus was reached.

### 2.6 Rigour and Quality Criteria

The interview guide was reviewed by a subject matter expert and consumer to confirm alignment with the research question and clarity of the questions, supporting credibility. Dependability was strengthened by two researchers independently coding transcripts and resolving discrepancies through consensus discussions. Credibility was supported through verbatim transcription and the use of quotations to reflect the perspectives of LEAs and stakeholders [7].

### 2.7 Ethical Considerations

Ethical approval was obtained from the organisational research office [QA2025.02] and an academic human research ethics committee [2025/HE000349] for this evaluative study. As described, individuals were provided with detailed information about the study to inform their decision to participate. Verbal consent was obtained before each interview. One LEA with an intellectual disability was potentially eligible for recruitment. To address associated ethical considerations, tailored support was implemented, including involvement of a Disability Liaison Officer during the consent and interview processes, provision of interview questions in advance, a low threshold for interview discontinuation if anxiety or distress were observed, and the use of a modified interview guide designed for low health literacy.

## 3 Findings

Fourteen individuals participated in this study, including all seven LEAs and seven stakeholders. Three stakeholders did not volunteer. Duration of interviews was 30 to 45 min, and nine interviews were online (e.g., six stakeholders, three LEAs) and five in‐person (e.g., one stakeholder, four LEAs). The LEA participants had been involved in a broad range of organisational projects to improve processes and/or policy, such as evaluation of primary health care for people in custodial settings, redesign of youth drug health service platforms, development of an overnight stay programme for carers, evaluation of community access to care following emergency presentation and equitable access to maternity care for people with disabilities. In addition, LEAs were employed across departments and services to contribute to development and/or enhancement of consumer guidelines, educational materials and cultural safety initiatives. Stakeholders were ward or departmental managers of various departments or services across the organisation.

Three key themes were identified as shown in Table 2: ‘Preparation for the LEA role’, ‘Valued contributions to organisational projects’, and ‘Restrictions on role influence’.

**Table 2:** Key themes and related sub‐themes.

Preparation for the LEA role	Importance of comprehensive orientation, onboarding and training
	Clarity about role description
	Building an LEAP team
Valued Contributions to Organisational Projects	Building relationships for project duration
	Positive impact on change
	Commitment to building consumership
Restrictions on role influence	Limited working hours
	Lack of career progression
	Genuine engagement by clinicians and managers

Abbreviations: LEA, lived experience advisor; LEAP, lived experience advisor programme.

### 3.1 Preparation for the LEA Role

Identified as a key strategy for successful implementation, participants expressed the importance of LEAs being adequately prepared for their new role. Three sub‐themes were identified: ‘Importance of comprehensive orientation and onboarding’, ‘Clarity about role description’, and ‘Building an LEAP team’.

### 3.2 Importance of Comprehensive Orientation and Onboarding

There was acknowledgement by LEAs about their experience of orientation and onboarding. Some recalled being introduced to healthcare staff they would be working with on specific projects. Orientation to the environment was also described, however, some participants explained they were only exposed to one campus on commencement: the study site has five hospital sites.

When they were onboarding, we did … inductions and everything to [one] campus. Then … we get the first booking at [another hospital]. They were very confused, … although we mentioned that … [as] part of their role, they need to attend different … campuses across [the health service].

[SH 1]

There were inconsistencies in onboarding processes amongst LEAs. While some reported completion of a comprehensive induction, others experienced minimal guidance:

I didn't really receive any orientation. I started at the same time as someone else, and we had a session, but it was more about where to get your security pass and some online education stuff.

[LEA 6]

Consideration of disability and special needs for LEAs was cited as an important consideration. They described that newly employed LEAs are likely to need additional support and adjustments as new employees as they have had a lived experience of poor health, disability and/or trauma.

We need to do work around their access needs and ways of working and … how we recruit these people. Because, depending on what their lived experience is, if it's from a disability perspective, have we got the system set up so that we can actually … recruit these people in a way that's accessible to them?

[SH 4]

It would have been better to have an option to have an appointment with someone [for] onboarding … There's a lot of information to process, and the requirements about immunisations … was very overwhelming. There was nothing in the process that adjusted for the fact that [you have a] lived experience. … We're all suffering some version of trauma and/or disability … We can become more overwhelmed by things. For us to process the information well, we need supports and adjustments. You're not hiring a neurotypical, abled, calm, non‐stressed person.

[LEA 5]

Appreciation for provision of education and training was expressed by LEAs, particularly when this advanced their knowledge for planned project duties. Opportunities to enrol in professional workshops and online learning modules were highly valued.

I've really enjoyed some of the trainings and workshops that have been offered to me. … I was really fortunate to participate [in] the Dementia Australia workshop where we had a virtual reality exercise, which was really empowering for me.

[LEA 1]

I do feel like the training was very thorough. I really enjoyed doing the modules related to diversity, equity and inclusion.

[LEA 2]

Some participants also reported that more training may be needed in consideration of different portfolios. In particular, writing and leadership skills were seen as essential LEA competencies.

When we are paying for someone to be part of a project, it's not just about providing the voice of the consumer. It's also about the administrative content. … ‘How do you write that in a professional sense … when you're not a professional?’ … A part of that role really is about … building those skills.

[SH 2]

I feel like there might be some other areas that could … be incorporated when … a lived experience worker is starting their role. … Like training on storytelling. … I couldn't find that as a resource. … It would be important for LEAs … to … have access to different leadership modules, as well as how to work with clinicians in a team, and … sharing our experience and expertise.

[LEA 2]

Getting the balance right between contributing important opinions versus providing too much information was highlighted as an area that LEAs may require support and training.

‘How do they represent a broad view versus their own view?’,… ‘How do you get someone with the right knowledge and experience … but can see a bigger perspective’. It's a balance …on how you get them to think broadly when they've got a lived experience.

[SH 5]

I think there're some gaps in our understanding of how we're supporting our consumers in the program. Some of the feedback … I've had from the LEA is that … they're worried that they're overstepping the mark. I'm just trying to encourage them to put their voice forward.

[SH 6]

Stakeholders discussed the difficulties LEAs had with writing documents.

I think the LEA is in such a different category … when it comes to writing a presentation or writing a report … Because an LEA doesn't necessarily have a university degree … and they're not necessarily coming with a whole lot of different work experience.

[SH 2]

### 3.3 Clarity about Role Description

Provision of a clear role description was highlighted as an important foundation for LEAs and healthcare staff. Some LEAs recalled feeling unsure about their responsibilities at first.

Having guidelines or a framework would be beneficial because they would [give] future workers of the program some structure, … knowing where to get the support …, what to do if something's not working … At the beginning I just felt like I was … on my own with dealing with everything.

[LEA 2]

I think one of the challenges I have … is they still haven't worked out what they want from me. … What I'm providing is not what they're wanting, but they're not telling me what they want.

[LEA 7]

Lack of role clarity was also highlighted by stakeholders. However, there was an appreciation that as a new position in the health service, understanding of the role would grow.

We're learning to work with an LEA. That's not a criticism or anything. ‘How does that look?’, ‘What does that role do?’, ‘What do they need to achieve?’ It's probably been the more burdensome part of the project … So understanding those expectations [and] building the role.

[SH 2]

I'm keen to learn … about how the actual program supports consumers to build their capability to work within the sector and within these programmes … I think that there [are] some gaps in our understanding of how we're working with lived experience.

[SH 6]

Mismatched expectations about the role compromised the potential value of the inclusion of an LEA in some projects. This led to frustration and confusion for LEAs and healthcare staff.

[One department] ran a project where I don't think they really knew what they wanted from the LEA … The … team didn't really seem to know what they wanted [the LEA] for … I don't think they used her to the best of her ability. We've used her in lots of projects and she's extremely productive and insightful.

[SH 4]

There's a couple of times where [the LEAs] expectations have been higher than what I need her to do. … There's been times when her suggestions have been clinical instead of just experience‐based… What she brings is so valuable but, sometimes, what she's bringing is too much and not about the project.

[SH 5]

Some concerns were also raised about clinicians' acceptance of the new role. Participants described that power imbalances may impact the full acceptance of LEAs as formal team members.

Some of the clinicians in our team … have found it a little bit challenging. … There's this historic cultural power imbalance around clinician and consumer … I also think there was just a bit of a cultural thing: … ‘Well I'm the clinician, so I know best and you're new’. That was really unfortunate to see.

[SH 4]

As clinicians, we're not always comfortable with that … It's creating that little bit of discomfort for … the greater good, because we're actually going to improve our services to meet the needs of consumers, not just what we think they need. … It's been a healthy disruption … It's going to frame the future for our clinicians and being comfortable with the consumer in the room and having those very transparent conversations.

[SH 6]

### 3.4 Building an LEAP Team

Participants discussed the importance of feeling connected with other LEAs. Opportunities to discuss their experiences and associated challenges were seen as essential in development in their new role. One participant described attending CoP meetings, organised by the LEAP manager.

I do also enjoy how… [the manager] runs community of practice meetings … All the LEAs [attend] when they can. … We talk about what's going on for each of us – any updates.

[LEA 2]

However, LEA participants raised concerns about the lack of opportunities to engage with other LEAs face‐to‐face.

I think that if you had a face‐to‐face meeting of all the LEAs in the same room, enormous things could happen … I went to a coffee and conversation thing, where I got to meet another LEA and had a lovely conversation with her … But that was the first … and only conversation I've had with her face to face.

[LEA 6]

Several stakeholders also raised the possibility of their attendance at CoP meetings to increase their understanding of the role, and to meet all LEAs. In their view, this would provide more flexibility in terms of employment of LEAs for their projects.

If the roles are going to be outsourced to other sites or programmes, either a community of practice or a meeting where you could have a chat to the [manager], … just to work through … issues … and any other updates. That's going to improve my learning as a manager. … If I met one of the LEAs, and go, ‘They might be also really well suited to be part of our team, or we could utilise them for this.’

[SH 7]

### 3.5 Valued Contributions to Organisational Projects

Participants expressed the view that the LEAP offered significant value to organisational projects. Three sub‐themes were identified: ‘Building relationships for project duration’, ‘Positive impact on change’, and ‘Commitment to building consumership’.

### 3.6 Building Relationships for Project Duration

Participants described that sustained engagement by LEAs for the duration of a project strengthened group relationships. This empowered LEAs to contribute to important discussions.

They built that rapport slowly with the team that they worked together with. After a while, they seemed to be quite enjoying [it], [integrated] with the team very well, lots of good conversations, laughs going around when they came in.

[SH 1]

… the LEA role has a bit more continuity [for] working with a team. It's going to be a set time, so two years. … That is a bit more time to get to know people well and develop better working relationships with them.

[LEA 2]

One participant described her feelings when contributing to project work. Involvement from the start of the project increased her engagement.

When I've been involved in processes early enough so that I can … offer comments that can be digested and become of use … I really enjoy being able to … get the sense of the whole thing. … Those comments were then received with ‘Oh, they were cool comments. We haven't thought of this, that, or the other either’. It's early enough for them to consider whether it's appropriate.

[LEA 5]

Contributions by LEAs to the project work were valued by stakeholders.

Rather than [talking] to the consumer separately, … having an LEA directly involved … makes everyone sit up a bit straighter and really think about what we're asking and what is important, rather than having side conversations with the consumer.

[SH 2]

They also described the reduction of administrative burden compared with identifying and recruiting individual consumers for each project.

For big projects like ours, it makes involving a consumer more formal, and almost simplifies the process, because you're not reaching out on an ad hoc basis. That person is embedded in the team. It makes … for more efficiencies … for both us and for the LEA.

[SH 2]

### 3.7 Positive Impact on Change

Overwhelmingly, participants described that the LEAP had led to improvements in healthcare provision within the organisation. In their view, these improvements were initiated through opinions shared by LEAs in different projects.

I think it is a really crucial and valuable role within spaces where people who have been able to experience that sort of environment, be a voice in making something more effective and more centred, … [it] is one of the biggest ways to create positive change. … It's not just about the people that work there, but also about the people who have experienced it.

[LEA 1]

Sharing lived experiences of healthcare was seen as an impactful opportunity to offer consistent value to the organisation. Inclusion of a consumer in project teams fostered open and transparent conversations about health service improvements.

LEAs, … the service users of the [organisation], will give … firsthand feedback about the service, how they would like to be treated … to the workforce. … They have that experience from their day‐to‐day living. To put that experience into the practical aspect of the service delivery, … that's really crucial and important for the continuous improvement of our service.

[SH 1]

Our LEA … will make one statement that is so all‐encompassing. It really makes everyone pause and reflect and reminds everyone what is important. … [She] doesn't have to say much but always gives people the opportunity to pause and really think about what we're doing, and why we're doing what we're doing.

[SH 2]

LEAs recalled that their contributions were valued in project meetings. Some reported that this led to important changes or developments in practice and/or procedure.

I'm in meetings where … I'm actually making suggestions and being like, ‘Look, I don't think that adds any value. Have you guys thought about how many places this report is going to get sent, and how many people are going to read it? All those people don't need to know X, Y, and Z.’ … It feels a lot more two‐way.

[LEA 5]

I like being staff. I like being a grown‐up who's getting paid. I like the accountability. I like the community and connection. I like the organic ideas being produced within the … team. … Being part of that brain pool. … None of that sort of stuff would happen as a consumer.

[LEA 5]

Some examples were provided by participants regarding specific projects in which it was perceived that LEAs played an important role in policy/procedure change.

We had an LEA in that project team. [It] was really important … to have that voice. … That pushed the project team … to change their way of working with consumers and carers. … Hearing how they changed their approach from what would have been a very … risk‐adverse [approach] … to a much more person‐focused approach, that was a big tick for us early on in the program.

[SH 4]

So we've moved away from a brochure … to something that's quite youth friendly and … lots of pictures. … The [LEA] provided that insight … of what a young person would look for, or need, when they're accessing a service. … It's really important to be able to have someone that can represent young people in this space.

[SH 7]

In their view, inclusion of an LEA in project teams was a valuable chance to improve consumers' experience of health provision.

By embedding an [LEA] …, you're not just getting top‐down, you're getting bottom‐up as well. So, they're getting influenced from both sides, which enables a mindset shift quicker … You're working with the staff who are working in that system, and you are seeing how they navigate their system and where there's potential opportunity to improve that system to get better outcomes for consumers.

[LEA 3]

… they make sensible suggestions that sometimes clinicians overthink and overlook. They really bring service delivery or projects back to what is best for the … consumer … Their suggestions are always a lot more person‐centred. …. Clinicians … try to be person‐centred but ultimately, they're not really. They're really thinking through a very different lens. … a different approach.

[SH 4]

Inclusion of LEAs who have an intellectual disability, neurodivergence or social disadvantage was seen as a valuable opportunity to gain an understanding of healthcare provision from a different perspective.

The … training pack … was developed in conjunction with [an] LEA, someone with [an intellectual disability]. … It's a codesign with us and the person who's living with [an intellectual disability]. … I can see … their expectations when they come to the health service. … It gives us insight, … where we need to improve, how we need to treat people, how we need to communicate with those people with … an intellectual disability.

[SH 1]

We've used LEAs with lived experience of different disabilities or neurodiversity to … create programmes [and] training. … They're coming at it from a very different perspective than the clinicians in the team who are trying to develop it from a clinical perspective.

[SH 4]

We're doing some work in the autism space, and our LEA has been very active…. We've made some really solid improvements. We've got some good data to show that … having the LEA at all levels of … consultation and testing and implementation … has been vital to its' success, because there's things that we would never have thought of changing or adapting if we didn't have LEAs.

[SH 6]

Personal benefits for the individual LEAs were also identified, particularly self‐worth: ‘I love to keep my mum so proud of me and so supportive.’ [LEA 7] Another participant also discussed the social role that the organisation played in creating employment for individuals with disabilities.

Creating roles for people that can't currently fit in the mainstream system. I'm a valuable member of society, but because of my disability, because of my life circumstances, I can't do a standard full‐time … or part time job. But I have this value that I can bring to society, and I have a right to be employed and contribute to society … The concept of an LEA creates a space where I can contribute to society and earn an income.

[LEA 5]

### 3.8 Commitment to Building Consumership

There was recognition from participants that the organisation is building a strong culture of consumership. Various activities were cited in which LEAs were given an opportunity to lead.

Our LEA delivered a presentation to [the organisation's] consumers about … the project … That was really powerful,. … far more valuable … than having a project manager stand up and present… The consumers saw that we were really, truly interested in what they were saying. We weren't standing up the front ticking a box.

[SH 2]

We've got an LEA now in a capital project, which is virtually unheard of, and advising from the perspective of a consumer in the design of a hospital. Normally, the architects don't want to hear that; they just want to do what they think is best.

[SH 4]

### 3.9 Restrictions on Role Influence

Despite very positive opinions from participants regarding the LEAP, some barriers to role influence within the organisation were identified. Three sub‐themes were found: ‘Limited working hours’, ‘Lack of career progression’, and ‘Genuine engagement by clinicians and managers’.

### 3.10 Limited Working Hours

Participants described that the most significant barrier to utilisation of the LEAP was restrictions on employment hours. Most LEAs are employed one day per week or fortnight. Stakeholders explained that these limited employment hours restrict the involvement of LEAs in projects, reducing capacity for meaningful contributions to the project.

I think more [employment hours] definitely would improve the program delivery. … To have them more frequently, or an increased number of hours, would definitely help.

[SH 1]

Having one day a week … makes it really hard when you have meetings that are relevant for the LEA … across the week. So it relies on the LEA having flexibility, but that's not fair on the LEA.

[SH 2]

LEAs also expressed some frustration at not being able to engage in allocated portfolios more frequently due to their restricted employment hours.

My role is one day a week, I feel like the project says all these things that they want to achieve, and I feel like sometimes they're missing the mark. Because I know for a fact that there could be so many other things that I'm doing as extra things on top of that. But I just can't.

[LEA 2]

Having more funding so that we can have more hours because it's incredibly important work and there's not enough hours in the fortnight to do what we do. Sometimes some tasks get left behind a little bit because other tasks come up when there's no time.

[LEA 4]

One stakeholder proposed employment of a team of LEAS that could be allocated projects according to organisational demand.

Ultimately, I would … prefer to have a team of permanent staff who are lived experience staff that can attend projects and service improvement things as a group … I would … be recruiting people based on a variety of characteristics that are lived experience and … deploy[ing] the right people into projects rather than what we're doing at the moment, which is recruiting to a project with one role.

[SH 4]

### 3.11 Lack of Career Progression

Whilst LEAs generally reported a high level of work satisfaction in their role, they raised concerns about future career opportunities. They discussed that there was no clear career progression, and this led them to question their future employment within the organisation.

It's basically a fixed‐term contract with low hours. I don't know how much career progression there is … [There is] … so much uncertainty … at the moment … If a fulltime job opportunity comes up, I'm just going to take it.

[LEA 2]

One stakeholder proposed the development of a formal training programme as a recruitment strategy.

We need to look at our training pathways and create that career progression … ‘Is there a way that we can partner with universities … to provide placements for this workforce?’ Like graduate type programmes rather than just expecting people to be able to come in off the street and do the role.

[SH 4]

### 3.12 Genuine Engagement by Clinicians and Managers

The LEAs raised some concern that not all healthcare staff engaged with the LEAP in a genuine manner. Several LEAs describe being treated as a box‐ticking exercise or being overlooked. Despite allocation to specific projects, some LEAs explained that they were not always included in team activities. This diminished their perception of being a valued team member.

Members of the project team … [conducted] a visit to an external hospital, and this would play into some of the design features [of a new Hospital]. I was not invited … I feel … that wasn't very inclusive.

[LEA 2]

Some individuals also explained that their contributions were not always appreciated or acknowledged. They considered these experiences as demoralising and patronising.

They got me to review [a concept]. I … tried to do it as thoroughly as I could and came up with … really good suggestions. It was just like, ‘Yeah, okay, thanks. We'll just push that aside too.’ So, it was very demoralising.

[LEA 5]

As an LEA, I'm always the last to know about things. I provided some edits … on things where … mistakes were made, and there was no response … I have encountered tokenism.

[LEA 6]

Some stakeholders suggested that the organisation needs to consider strategies to improve involvement of LEAs. Most feedback focused on building relationships within teams to be more inclusive of LEAs.

I think it's an exciting piece of work that needs to happen across all health services. [We] have a way to go in terms of how we build consumers into everyday business, … in educating the workforce around how to … feel comfortable working with consumers and how to understand the perspective of consumers and adapt that into the work that we're doing.

[SH 6]

I think where [the LEAs] are given a seat at the table, and where they're treated with respect and integrated into a team, on an even playing field, they do really well … It really depends on setting them up for success there.

[SH 4]

## 4 Discussion

The purpose of this study was to increase our understanding about LEAs and stakeholders' perspectives about the LEAP. Overall, there was widespread acknowledgement that the programme is valuable and contributes to quality improvement initiatives and project goals. However, there may be opportunity to strengthen the programme, particularly through building role clarity, teamwork and individual skills.

We identified that individuals employed as LEAs require support and guidance to build confidence and competence in their new role. Although all LEAs receive standard employee induction training (e.g., workplace health and safety, cultural awareness), they are not formally trained in consumer engagement. This finding resonates with the systematic review by Wiles et al [3] of 23 studies in which no consumers received formal engagement training, suggesting that their lived experience was adequate expertise for their employment responsibilities. A standardised onboarding process for new LEAs should be implemented, with hospital processes explained and objectives, timeframes, expected outcomes of the role, and a clear strategy for periods of decreased workflow periods outlined.

There is a need for a comprehensive role description as a guiding document for individuals commencing employment in this role, with emphasis on the skills and experience that support consumers to be effective partners in quality improvement initiatives in healthcare [11]. These include lived experience as a patient or carer, ability to reflect on their personal experience, capacity to communicate effectively with diverse audiences, and authentic storytelling. Whilst it should not be an expectation that a consumer will be competent in all of these areas, employers should know what roles the LEA may be suited to and provide training and/or support in these areas where deficits are identified [11]. However, according to Parham et al. [12], there is limited evidence of capacity‐building interventions focused on enabling consumers to serve as leaders, agents of change, and service improvers. In response, they have developed a Collaborative Pairs programme in which consumers and clinical leaders are paired to work together on a shared challenge. The findings of this current study also indicate a need for stakeholders to better understand the roles and expectations of the LEAP. Development and dissemination of a role description for LEAs is needed to address institutional barriers related to lack of understanding about role clarity by individuals holding leadership roles and likely to interact with LEAs. The position description should include clear accountabilities and guidance about development of individual and team work‐plans.

Examples were provided in this study about the impact that LEAs can have on influencing decision‐making in project meetings through storytelling. However, not all LEAs were identified as having this skill. According to the World Health Organization [13], ‘Stories are at the heart of human existence and people are hard‐wired to respond to them’. Storytelling can influence how people think about information that is conveyed to them. Our study suggests that LEAs may need additional training in storytelling to maximise their impact in project work. Also, leaders may need to utilise strategies to empower consumers and build their confidence in expression of their lived experience in a safe environment. In support of this proposal, capability building was identified as a key strategy for healthcare organisations in the Strengthening Consumer Engagement Report [14].

Whilst there were generally positive opinions about the integration of LEAs into the health workforce, concerns were raised about their retention due to restricted employment hours and limited contract options. Employment at this organisation is sustained by executive leadership funds at their discretion. To justify ongoing employment, measures of improved health or financial outcomes may be needed. This may be challenging, as to date, there is limited evidence that partnering with consumers improves service delivery [15, 16].

Of concern, partnership dynamics and processes identified in this study may restrict the impact of the LEAP, including perceived power imbalance. LEAs did not always perceive that their contributions were valued by the project team. This finding resonates with the systematic review by Merner et al. [16] in which a lack of clarity about the consumers' role can limit consumers' involvement and confidence. They also found that consumers can feel frustrated when they perceive tokenistic involvement. In their qualitative study, [17] identified that healthcare professionals have significant power over consumers in partnerships at the systematic level, and consumers did not feel like equal partners Merner et al. [16]. proposed that implementation of best practice principles for formal partnering (e.g., leadership and health service culture; diversity; equity; mutual respect; shared vision and regular communication; shared agendas and decision‐making; influence and sustainability) may help to address power imbalances and improve consumers' experience of formal partnerships.

### 4.1 Strengths and Limitations

A strength of this study is the inclusion of viewpoints from both stakeholders and LEAs, providing a comprehensive view of experiences. To reduce bias and promote more open responses, the researcher assigned to conduct the interviews and analysis had no pre‐existing relationship with the participants.

This study did not examine perceptions from all project team members regarding involvement of LEAs in their projects. Their experience may differ from stakeholders who mainly hold non‐clinical leadership roles. Hence, future studies might examine broader perspectives from other team members. It should also be noted that the research was conducted within a particular health service precinct, which may limit the generalisability of the findings.

### 4.2 Implications for Practice and Policy

Through paid remuneration as LEAs, the organisation in which this study was conducted has implemented a strategy that could be recognised as a bridge to improving the professional profile of consumers. Paid participation was a key strategy outlined by the National Mental Health Commission [18] in building consumership within health services. However, several findings require further consideration in the future development of the LEAP. To improve the perception by LEAs that their input is valued, healthcare professionals and managers should be encouraged to reflect on the effect that power has on their relationship with consumers [16] and provide consumers opportunities to speak and contribute in a meaningful way so that they feel valued (Schulz et al. 2018). Education providers could also ensure that curriculum addresses this issue.

The position description should be formalised, ideally, with input from experienced LEAs and organisational leaders with experience in consumer engagement. Prior to allocation of an LEA in specific projects, healthcare professionals should be provided with clear expectations about roles and responsibilities. Where needed, training should be provided to enable the LEA to confidently and competently participate. Further Loughhead et al. [19], proposed that guidance, processes and strategies are needed to genuinely transform the way in which lived experience leaders collaborate and work with healthcare project teams. In their action research study, a key learning was the importance of the need to create awareness about lived experience leadership, how it was defined and understood as well as its potential.

## 5 Conclusion

The findings of this study suggest that organisational commitment to increase consumership through employment of consumers with lived experience in health service planning is valued. Healthcare professionals and LEAs perceive that consumer involvement for the duration of projects has a positive impact. Clearer guidelines and role descriptions, and formal training initiatives for consumers and healthcare professionals, are needed to promote the LEAP to specifically address perceived power imbalances within these partnerships.

## Author Contributions

**Rebecca Barbara:** conceptualisation, investigation, writing – original draft, methodology, writing – review and editing, formal analysis, resources. **Rohan Vaughan:** conceptualisation, methodology, writing – review and editing, resources. **Joanna Petrunic:** investigation, writing – review and editing, formal analysi, project administration, data curation. **Debra Kerr:** conceptualisation, investigation, writing – original draft, methodology, writing – review and editing, software, formal analysis, project administration, data curation, supervision.

## Funding

The authors have nothing to report.

## Ethical Statement

Ethical approval was obtained from the organisational research office for this evaluative study: Project ID 114554, Review Reference QA/114554/WH‐2025‐472166(v2). Individuals provided their voluntary, verbal consent for their participation in this study.

## Conflicts of Interest

The authors declare no conflicts of interest.

## Supporting information


Supporting File

## Data Availability

Research data are not shared. Data will not be made available externally relating to ethics approval restrictions.

## References

[1] D. McGowan , C. Morley , E. Hansen , K. Shaw , and T. Winzenberg , “Continuity Is Essential: The Experiences of Co‐Design Participants During the Implementation of a New Health Service,” Health Expectations 28, no. 5 (2025): 1–12, 10.1111/hex.70436. PMC12446999 40969130

[2] C. Morley , K. Jose , S. E. Hall , et al., “Evidence‐Informed, Experience‐Based Co‐Design: A Novel Framework Integrating Research Evidence and Lived Experience in Priority‐Setting and Co‐Design of Health Services,” BMJ Open 14, no. 8 (2024): e084620, Published 2024 Aug 8, 10.1136/bmjopen-2024-084620. PMC11404138 39122385

[3] L. K. Wiles , D. Kay , J. A. Luker , et al., “Consumer Engagement in Healthcare Policy, Research and Services: A Systematic Review and Meta‐Analysis of Methods and Effects,” PLoSONE 17, no. 1 (2022): e0261808, 10.1371/journal.pone.0261808. PMC8794088 35085276

[4] A. E. Hall , J. Bryant , R. W. Sanson‐Fisher , E. A. Fradgley , A. M. Proietto , and I. Roos , “Consumer Input Into Health Care: Time for a New Active and Comprehensive Model of Consumer Involvement,” Health Expectations 21, no. 4 (2018): 707–713, 10.1111/hex.12665. 29512248 PMC6117488

[5] R. Barbara , J. Lydeker , A. Potter , and D. Kerr , “Lived Experience Advisor Program Initiative: Harnessing Consumer Leadership for Best Care,” Australian Health Review 49: AH24311, 10.1071/AH24311. 39894016

[6] Australian Commission on Safety and Quality in Health Care . Partnering With Consumers Standard. Australian Commission on Safety and Quality in Healthcare. 2019 www. safetyandquality.gov.au/standards/nsqhs‐standards/partnering‐consumers‐standard [cited 6 September 2024].

[7] M. A. Neergaard , F. Olesen , R. S. Andersen , and J. Sondergaard , “Qualitative Description ‐ the Poor Cousin of Health Research? *Biomed Central Medical Research Methodology* ,” 52 9, no. 1 (2009), 10.1186/1471-2288-9-52. PMC2717117 19607668

[8] A. Tong , P. Sainsbury , and J. Craig , “Consolidated Criteria for Re‐Porting Qualitative Research (COREQ): A 32‐Item Checklist for Inter‐Views and Focus Groups,” International Journal for Quality in HealthCare 19, no. 6 (2007): 349–357, 10.1093/intqhc/mzm042. 17872937

[9] B. Saunders , J. Sim , T. Kingstone , S. Baker , et al., “Saturation in Qualitative Research: Exploring Its Conceptualization and Operationalization,” Quality & Quantity 52, no. 4 (2017): 1893–1907, 10.1007/s11135-017-0574-8. 29937585 PMC5993836

[10] V. Braun and V. Clarke , “Using Thematic Analysis in Psychology,” Qualitative Research in Psychology 3, no. 2 (2006): 77–101, 10.1191/1478088706qp063oa.

[11] Health Issues Centre . 2023. Consumer Model – Partnering with healthcare organisations.

[12] J. Parham , A. Harvey , L. Wells , J. Cockburn , and K. Patterson , “Building Collaborative Practice With Consumers in Rural and Remote Australia,” 15th National Rural Health Conference (2019): 24–27 March.

[13] World Health Organization . The power of storytelling for health impact. 2024, www.who.int/westernpacific/newsroom/feature-stories/item/the-power-of-storytelling-for-health-impact.

[14] Commission on Excellence and Innovation in Health . Strengthening Consumer Engagement. December 2022, ceih.sa.gov.au/assets/library/Strengthening-Consumer-Engagement-in-Healthcare.pdf.

[15] D. Lowe , R. Ryan , L. Schonfeld , et al., “Effects of Consumers and Health Providers Working in Partnership on Health Services Planning, Delivery and Evaluation,” Cochrane Database of Systematic Reviews, no. Issue 9 (2021): Art. No: CD013373, 10.1002/14651858.CD013373.pub2. PMC8440158 34523117

[16] B. Merner , L. Schonfeld , A. Virgona , D. Lowe , L. Walsh , et al., “Consumers' and Health Providers' Views and Perceptions of Partnering to Improve Health Services Design, Delivery and Evaluation: A Co‐Produced Qualitative Evidence Synthesis,” Cochrane Database of Systematic Reviews (2023, Issue 3. Art. No.: CD013274), accessed 16 March 2026, 10.1002/14651858.CD013274.pub2. PMC10065807 36917094

[17] B. Scholz , J. Bocking , C. Platania‐Phung , M. Banfield , and B. Happell , “Not An Afterthought”: Power Imbalances in Systemic Partnerships Between Health Service Providers and Consumers in a Hospital Setting,” Health Policy 122, no. 8: 922–928, 10.1016/j.healthpol.2018.06.007. 30017107

[18] National Mental Health Commission ., Paid Participation Policy for People With a Lived Experience of Mental Health Difficulties, Their Families And Support People (National Mental Health Commission, 2017).

[19] M. Loughhead , E. Hodges , H. McIntyre , et al., “Pathways for Strengthening Lived Experience Leadership for Transformative Systems Change: Reflections on Research and Collective Change Strategies,” Health Expectations 27, no. 5 (2024): e70048, 10.1111/hex.70048. 39361254 PMC11447884

